# Suppression of Obsessive-Compulsive Symptoms after Head Trauma

**DOI:** 10.1155/2012/909614

**Published:** 2012-08-26

**Authors:** Seyed Hamzeh Hosseini, Paria Azari, Roohollah Abdi, Reza Alizadeh-Navaei

**Affiliations:** ^1^Psychiatry and Behavioral Science Research Center, Mazandaran University of Medical Sciences, Sari 48154-66848, Iran; ^2^Department of Radiology, Mazandaran University of Medical Sciences, Sari, Iran; ^3^Deputy of Research, Mazandaran University of Medical Sciences, Sari, Iran

## Abstract

Obsessive-Compulsive Disorder (OCD) encompasses a spectrum of clinical symptoms characterized by unwanted thoughts coupled with an intense compulsion to act and to repeat behavior fragments in a ritualistic and stereotyped sequence. Obsessive-compulsive symptom due to brain lesions is not rare, but suppression of these symptoms after head trauma is very rare and we found only 3 cases in review of literatures from 1966 to 2001. The case of a patient suffering with severe OCD is described of note; her symptoms disappeared following right temporo-parietofrontal lesion.

## 1. Introduction

The cardinal manifestations of OCD are recurrent intrusive, ego-dystonic thoughts, images, and urges portending adverse consequences leading to feelings of anxious dread, patients therefore engage in mental and motor compulsions (repetitive ritualized thoughts or acts) in an attempt to neutralize the obsession/adverse consequence. The obsessions or compulsions are time consuming and interfere significantly with the person's normal routine, occupational functioning, usual social activities, or relationship [1]. 

### 1.1. Brain-Imaging Studies

Neuroimaging in OCD patients has produced converging data implicating altered function in the neurocircuitry between orbitofrontal cortex, caudate, and thalamus. Various functional brain-imaging studies—for example, positron emission tomography (PET)—have shown increased activity (e.g., metabolism and blood flow) in the frontal lobes, the basal ganglia (especially the caudate), and the cingulum of patients with OCD. The involvement of these areas in the pathways with the amygdale pathway that are the current focus of anxiety disorder research. Pharmacological and behavioral treatments reportedly reverse these abnormalities [2].

In a normal subject, the orbitofrontal cortex would stimulate the caudate, which, in turn, would stimulate the pallid nucleus, consequently inhibiting the medial thalamic nucleus, the main station to the frontal cortex. The medial thalamic nucleus would modulate and correct the hyperactivity of orbitofrontal thalamic stations. Dysfunctions in the modulatory activity of these interconnections may possibly be present in patients with OCD and hyperactivity of the thalamic-orbitofrontal interconnection may produce obsessive thoughts and compulsive rituals [3].

According to this scheme striatal dysfunction leads to inefficient thalamic gating, resulting in hyperactivity within the orbitofrontal and cingulate cortex. This could result in the intrusive cognitive phenomena and the associated anxiety. Extending this of thought, compulsions could be conceptualized as repetitive mental and motor behaviors, performed to recruit the striatum so as to ultimately achieve thalamic gating transiently neutralizing the anxiety and thoughts [4].

 The most common psychosurgical procedure for OCD is cingulotomy, which is successful in treating 25 to 30 percent of otherwise treatment-unresponsive patients [2].

## 2. Case Presentation

**Figure 1:**
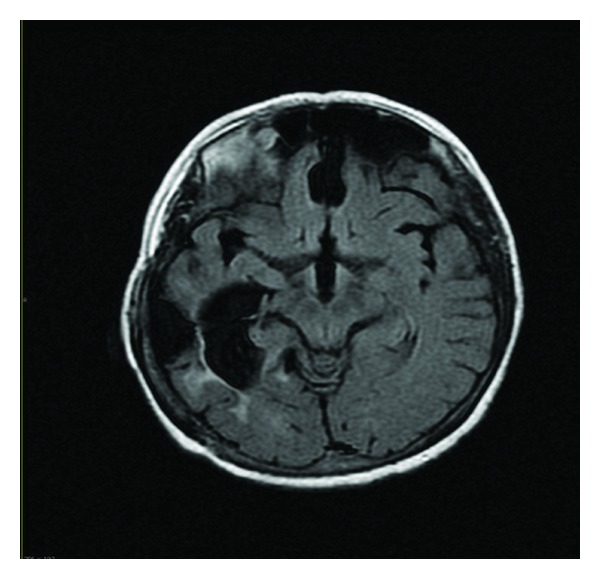
Axial FLAIR image.

The patient was a 44-year-old female, who has suffered from contamination and pathological doubt obsessions and washing and checking compulsions from adolescence. The score of Yale-Brown scale was thirty. The disorder had deteriorated her social and interpersonal relations, but prior to this accident, she did not take any treatment for obsessive-compulsive disorder. In a car accident, a temporoparietal hemorrhage occurred, and she was in coma state for 3 months. After this period, her symptoms disappeared. The score of Yale-Brown scale has been zero yet. MRI images of the brain were performed few days of initial episode of injury showed a temporoparietal hemorrhagic contusion. In followup, after 9 years, MRI showed focal atrophy of the temporal lobe with associated dilatation of the temporal horn of the right lateral ventricle (Figure 1). There are multiple foci of high signal intensity within the subcortical and deep cerebral white matter in the parietal, frontal, and temporal lobes. The patient has not any cognition problem, and minimental state score is thirty now.

## 3. Discussion

Studies that investigate the pathophysiology of OCD are therefore of considerable importance, because they may ultimately inform treatment strategies. Functional brain imaging in obsessive-compulsive disorder demonstrated decreased blood flow in the temporal lobes as well as cortical perfusion abnormalities in the frontal lobes abnormal blood flow may be seen in a number of different brain regions in acquired OCD. It is unclear whether these changes reflect primary neurological lesions or secondary changes to compensate for such damage. However, increased frontal blood flow in OCD may be hypothesized to reflect a compensatory mechanism [5]. Neuroimaging studies consistently associated dysfunction with prefrontal cortico-striatal-thalamic-cortical (CSTC) pathways in obsessive thoughts and compulsive urges [6]. According to this scheme striatal dysfunction leads to inefficient thalamic gating, resulting in hyperactivity within the orbitofrontal and cingulate cortex. This could result in the intrusive cognitive phenomena and the associated anxiety. Extending this of thought, compulsions could be conceptualized as repetitive mental and motor behaviors, performed to recruit the striatum so as to ultimately achieve thalamic gating transiently neutralizing the anxiety and thoughts [4]. It is noteworthy that the orbitofrontal cortex might play a potential role in inhibitory motor control. This may be of special importance given the compulsions presented in OCD and the patients; attempts to resist performing these actions [7].

Our review of Medline from 1966 to 2001 showed that our patient is the 4th case of OCD whose symptoms significantly disappeared following head trauma. One is a patient with severe obsessive illness who sustained a severe head injury. As a consequence of his injury, the patient developed a left prefrontal subdural haematoma. Upon his recovery, the obsession symptoms disappeared [8]. The other case is a man who attempted suicide by shooting his head over the left temple, causing left frontal lobe damage. Although no frontal lobe syndrome emerged, his obsessive rituals were significantly reduced [9]. The other case following the clinical diagnosis of arterial hypertension he sustained a brain hemorrhage. As a consequence of this cerebral accident his obsessive-compulsive symptoms were absent. However, as a progression of the clot retraction began gradually to take place, his obsessive-compulsive symptoms considerably returned [10]. The connection of these three cases, associated with OCD, indicates that specific cerebral sites affected by a given lesion may ablate “therapeutically” a preexisting disorder that is OCD. Furthermore, because in one case lesions are located in the left basal ganglia region, the other two in the left frontal region, and in our case, lesions located in temporoparietoccipital. Suggests that lesions affect association circuits linking neuronal columns of the BG and the prefrontal area, regardless of a specific anatomical location within the operational circuit, may modify obsessive-compulsive symptomatology.
